# Correlation analysis of osteoporosis and vertebral endplate defects using CT and MRI imaging: a retrospective cross-sectional study

**DOI:** 10.3389/fphys.2025.1649477

**Published:** 2025-09-23

**Authors:** Song Hao, Luo Yuxiao, Li Huilan, Hua Jun, Liu Dong

**Affiliations:** ^1^ Department of Orthopaedics, Hospital Affiliated to Hangzhou Normal University, Hangzhou, Zhejiang, China; ^2^ Department of Orthopaedics, The Second Affiliated Hospital of Soochow University, Suzhou, China; ^3^ Suzhou High-Tech Zone Yangshan Community Health Service Center, Suzhou, China

**Keywords:** osteoporosis, bone mineral density, vertebral endplate defects, HU value, MRI

## Abstract

**Background:**

Osteoporosis (OP) and vertebral endplate defects are important manifestations of vertebral degenerative changes that greatly affect the quality of life of elderly people. This study investigated the potential association between vertebral endplate defects and osteoporosis using imaging modalities.

**Methods:**

Computed tomography (CT), magnetic resonance imaging (MRI), bone mineral density (BMD) and other relevant imaging data, as well as age, sex, body mass index (BMI), and degree of low back pain data, were retrospectively analysed. The vertebral Hounsfield unit (HU) value and the maximum width and maximum depth of the vertebral endplate defect were measured and standardized. A HU <110 was defined as OP. Logistic regression was used to identify the risk factors for vertebral endplate defects.

**Results:**

Demographic data from a total of 199 patients were included in this study, along with data from 995 vertebral bodies. The relationships between the HU value and other data between the vertebral body defect group and the nonvertebral body defect group were compared. We found significant differences in age (70.6 ± 8.4 vs. 63.8 ± 9.5, p < 0.001), sex (male/female) (26/69 vs. 43/61, p < 0.05), BMI (23.8 ± 3.4 vs. 24.8 ± 3.4, p < 0.05), and total spine HUs (84.65 ± 35.49 vs. 124.86 ± 49.59, p < 0.001). The lower HU group had larger endplate defects (p < 0.001, p < 0.01), and the lower endplates had a greater standardized defect width and cumulative defect score than the upper endplates (p < 0.01). There were statistically significant associations between endplate defects and age (OR = 1.0, p = 0.042) and total spine HUs (OR = 0.98, p = 0.001).

**Conclusion:**

There was a correlation between OP and the size of the vertebral endplate defect, and the defect size increased with decreasing bone mass. According to our results, vertebral endplate defects are more likely to occur in elderly individuals, females, and individuals with OP. With respect to the spinal structure, vertebral endplate defects are more likely to occur in the upper lumbar spine. Age and bone mass are the main factors associated with vertebral endplate defects.

## Introduction

Osteoporosis (OP) is a degenerative disease characterized by reduced bone mass and bone structural degeneration, which leads to changes in the bone microarchitecture and imbalances in bone homeostasis. OP is often closely related to bone pain and fragility fractures (Akkawi and Zmerly, 2018). In China, the prevalence of OP in women over 50 years old is 29%. In the United States, an estimated 10.2 million people 50 years or older have OP (Black and Rosen, 2016), and it is estimated that by the end of 2025, the economic burden of OP will reach USD 25.3 billion in the United States alone (Johnell and Kanis, 2006).

The vertebral endplate is a thin layer of tissue located on both sides of the intervertebral disc composed of cartilage and bone. The vertebral endplates constitute the mechanical interface between the intervertebral disc and the vertebral body and facilitate the even distribution of compressive stress across the vertebral body. In addition, the endplates contain bone marrow contact channels, which provide nutrients to the intervertebral discs, excrete metabolic wastes by diffusion, and enable the flow of fluid under cyclic loading (Holm et al., 1981; Mattei, 2013). Endplate defects can promote communication and continuous cross-talk between the intervertebral disc and vertebral body, which may lead to a persistent inflammatory state in the vertebral marrow and become an important source of low back pain. The relatively thin and perforated physiological structure of the vertebral endplate cannot bear the long-term compressive load and is prone to fracture and defect. Currently, some of studies have confirmed the connection between osteoporosis and endplate injury (Ferrar et al., 2007; Ferrar et al., 2012; Fujiwara et al., 2019; Homminga et al., 2001; Lentle et al., 2021; Wang et al., 2019; Zhang et al., 2021). With respect to whether OP further aggravates vertebral endplate destruction by affecting the bone microenvironment and increasing the fragility of the endplate, only a few studies have reported the relationships among these diseases. Xiao et al. reported OP-induced abnormal porosity in the cartilage endplates and harmful effects on the endplates caused by OP (Xiao et al., 2018). Wang et al. reported that vertebral endplate injury occurred in OP model rats (Wang et al., 2014). The pathogenic relationship between OP and vertebral endplate injury remains unclear.

As a mature bone mineral density (BMD) evaluation technology, dual-energy X-ray absorptiometry (DXA) is used in clinical diagnosis, but many studies have confirmed that vertebral hyperplasia, facet joint sclerosis, scoliosis, osteophyte and pannus formation can cause DXA measurement errors (Xiao et al., 2018; Bland et al., 2022). Recently, many experts have introduced and recommended an alternative method of assessing BMD in patients via the Hounsfield unit (HU) value through computed tomography (CT). Studies have confirmed that there is a suitable correlation between local BMD and HUs measured via CT (Alawi et al., 2021; Lee et al., 2013; Schreiber et al., 2011; Silva et al., 2012; Zou et al., 2020; Xue et al., 2024), and measurement errors caused by vertebral hyperplasia and facet joint sclerosis are avoided (Leboff et al., 2022).

X-ray, CT, and magnetic resonance imaging (MRI) can all visualize the vertebral endplates. Among them, MRI is considered the most sensitive and effective way to detect subtle changes in the structure of the endplate. Therefore, MRI can better reveal different degrees of endplate injury.

Recent studies on the relationship between OP and vertebral endplate defects involve qualitative grading rather than accurate measurement. Moreover, recent studies have reported that OP is closely related to the total endplate (TEP) score of vertebral endplate defects (Zhuang et al., 2021); however, the size and morphology of the endplate defect often affect other phenotypes, such as the high-density zone (HIZ), disc displacement, and facet joint changes. (Li et al., 2023). In view of these limitations, it is necessary to further investigate the relationship between OP and the phenotype and distribution of vertebral endplate defects and to further understand how OP is involved in the process of vertebral degenerative diseases.

## Materials and methods

### Study participants

PASS version 15.0 (NCSS, Caseville, Utah, United States) was used to calculate the required sample size. The data of all inpatients in the Orthopaedic Department of our hospital from January 2020 to December 2024 were analysed. The inclusion criteria were complete clinical data, including sex, age, height, weight, CT scan of the thoracolumbar spine, MRI of the thoracolumbar spine, and BMD. In addition, low back pain scores were measured via a visual analogue scale (VAS) (Supplementary Figure S1) to evaluate the severity of low back pain. The exclusion criteria were previous lumbar surgery history; spinal tuberculosis; spinal tumours; scoliosis; new vertebral fractures; patients with a history of spinal infections and other nonspecific infections; congenital spine diseases; and severe underlying diseases, such as heart, craniocerebral, and acute and chronic diseases of the lung and kidneys; and patients with rheumatoid arthritis, ankylosing spondylitis and other immune system diseases and genetic diseases. Demographic data such as sex, age, weight, and body mass index (BMI) were recorded and analysed. We confirmed that all patients involved in the study provided written and verbal informed consent, which was approved by the institutional ethics committee, and that all the data were anonymous and confidential. This study protocol was approved by the Institutional Ethics Committee of the Second Affiliated Hospital of Soochow University (JD-HG-2024-019) and implemented in accordance with the principles of the 1975 Declaration of Helsinki.

### DXA

All patients underwent dual-energy X-ray absorptiometry (DXA) examination of lumbar spine (L1-L4) using Prodigy Pro Compact (GE Healthcare, United States). Obtain the lumbar bone mineral density (BMD) and T-score of the participants.

### Measurement of HUs in patients

**FIGURE 1:**
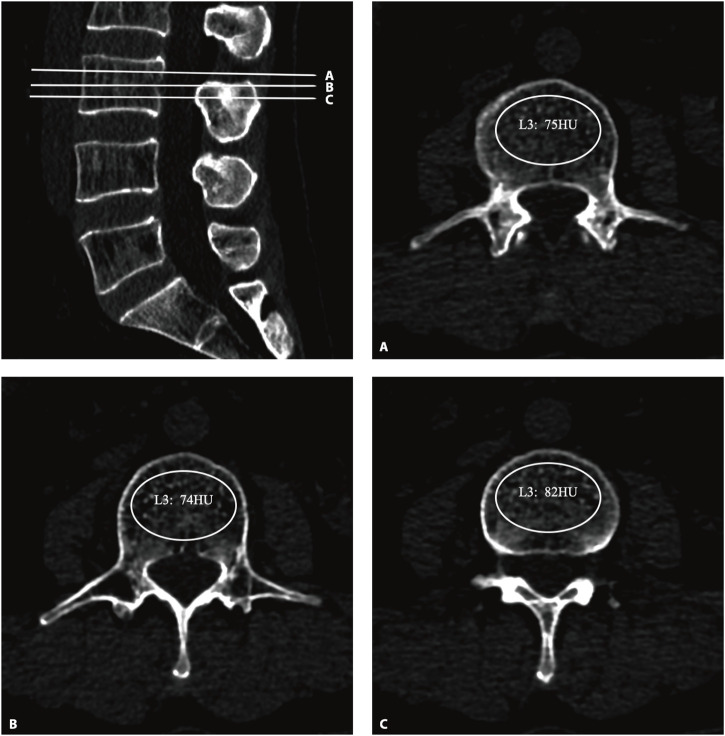
The HU values were derived from CT. An oval region of interest is positioned on the axial plane of the vertebrae **(A–C)**, with the HU value being automatically calculated by the image archiving and communication system. HU, Hounsfield unit.

All the subjects underwent three-dimensional (3D) lumbar CT (GE Optima CT660^−ΔΔCT^, United States), The parameters were as follows: tube voltage 120 kV; collimation, 64 × 0.625 mm; field of view (FOV), 500 mm; The CT scan data were transferred to the workstation and reconstructed with standard algorithms with reconstruction layer thicknesses of 1.25 mm, displaying a field of view of 380 mm; reconstruction matrix: 512 × 512. After the 3D CT image was reconstructed, the upper, middle, and lower planes of each vertebral body were selected to establish an elliptical region of interest (ROI) on the upper, middle, and lower planes perpendicular to the vertebral axis. The ROI was selected to include as much trabecular bone as possible and to exclude cortical bone, the posterior venous plexus, and bone islands (Figure 1). The mean of the HU values of the three ROIs for each vertebral body was recorded as the HU value of the vertebral body, and the mean of the HU values of L1–5 was calculated as the total spine HU value for this patient. According to previous studies, the diagnostic threshold of OP with high sensitivity and specificity was determined to be 110 HUs (Alawi et al., 2021; Xue et al., 2024; Zou et al., 2019; Pickhardt et al., 2013; Wagner et al., 2016).

### Endplate defect analysis and scoring

All the subjects underwent lumbar imaging with a 3T MRI scanner (Achieva, Philips Healthcare, Best, Netherlands). A standard spin echo imaging sequence was used for sagittal T2-weighted (T2W) MRI. The parameters were as follows: field of view, 30*20 cm, layer thickness 4 mm, acquisition matrix 400*232, and echo time/repetition time (TE/TR) 100 ms/4940 ms. Axial t1-weighted (T1W) MRI was also acquired via the following parameters: FoV 18*18 cm, layer thickness 4 mm, and TE/TR 10 ms/500 ms.

**FIGURE 2:**
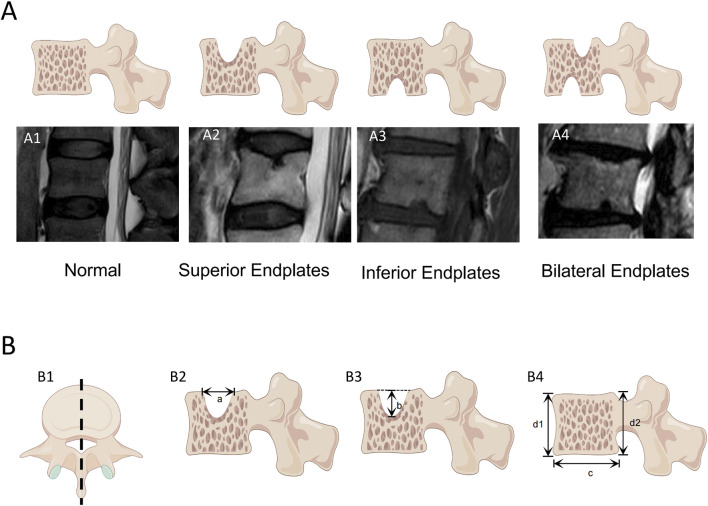
Measurement of vertebral endplate defects The endplate defects were measured in sagittal T2W weighted images of the vertebral body by a PACS system. **(A)** Classification of endplate defects: (A1) normal endplate; (A2) Superior Endplates Defect; (A3) Inferior Endplates Defect; (A4) Bilateral Endplates. **(B)** Measurement of vertebral body dimensions and endplate defect dimensions: (B1) Obtain a lateral view of the vertebral body along the central axis of the vertebral body; (B2) The maximum defect width of the endplate is measured to be a; (B3) The maximum defect depth of the endplate is measured to be b; (B4) Measure the width of the vertebral body as c, The height of the vertebral body was measured as (d1+d2)/2. The vertebral body measurement is based on the maximum width and depth of the vertebral body as captured in (B1).

T2W sagittal images of the L1‒L5 lumbar motion segment endplates of all the subjects were evaluated, and the vertebral segment and location of the defective endplate were recorded. For all the endplates with defects in the vertebral bodies, the maximum defect depth and maximum defect width on the sagittal images were measured. The width and height of the defective vertebral body were measured in the sagittal plane (Figure 2), which were used to calculate the normalized width and depth of the endplate defect. When more than two defects are observed, the width of the vertebral endplate defect is the accumulation of the widths of each defect, and the depth of the vertebral endplate defect is the accumulation of the depths of each defect. The ratios of width and depth of the endplate defect to the width and height of the vertebral body were calculated. The cumulative normalized endplate defect of a single vertebral body was determined by adding the normalized width and normalized depth of the endplate defects. In this experiment, the observers have more than 10 years of experience in classifying images.

### Statistical analysis

SPSS Statistics version 25.0 software (IBM Corp., Armonk, NY, United States) was used for data analysis. When the sample size is ≥50 cases, the Kolmogorov-Smirnov test is used for normality test; when the sample size is <50 cases, the Shapiro-Wilk test is used. The data with a normal distribution were expressed as mean ± standard deviation (SD). Intergroup analysis was performed via an independent samples t-test, and the non-normally distribution, Intergroup analysis was performed via Mann-Whitney U test. The χ ^2^ test was used for categorical data. Descriptive statistical methods were used to calculate the incidence, distribution, and location of endplate defects. Finally, univariate and multivariate logistic regression were used to verify the independence of the statistical results, and p < 0.05 was considered significant.

## Results

Table 1 summarize and compare the demographic characteristics of the 199 patients who met the inclusion criteria during the study period. Among them, 69 patients were males, and 130 were females. The mean age was 67.1 years (range: 39–87, SD: 6.6 years). The mean BMI was 24.4 kg/m^2^ (range: 16.2–36.4, SD: 3.5 kg/m^2^).

**TABLE 1:** The characteristics of the participants.

	Endplate defects	No endplate defects	Total	P value
	95(%)	104(%)	199(100%)	
Age, mean(SD), y	70.6 ± 8.4	63.8 ± 9.5	67.1 ± 9.6	<0.001
sex				0.039
female	69(73%)	61(59%)	130(65%)	
male	26(27%)	43(41%)	69(35%)	
Height mean(SD), cm	157.0 ± 8.2	160.6 ± 9	158.9 ± 8.8	0.004
Weight mean(SD), kg	58.9 ± 10	64.2 ± 11.2	61.7 ± 11.0	0.001
BMI, mean(SD)	23.8 ± 3.4	24.8 ± 3.4	24.4 ± 3.5	0.044
Total spine BMD, (g/cm2)	0.873 ± 0.159	1.018 ± 0.247	0.949 ± 0.221	<0.001
T value, mean(SD)	−1.6 ± 1.4	−0.2 ± 1.9	−0.9 ± 1.8	<0.001
Total spine HU, mean(SD)	84.65 ± 35.49	124.86 ± 49.59	105.67 ± 47.78	<0.001
L1 HU, mean(SD)	89.07 ± 35.58	129.61 ± 48.78	110.26 ± 47.44	<0.001
L2 HU, mean(SD)	82.51 ± 37.79	124.06 ± 50.34	104.22 ± 49.29	<0.001
L3 HU,mean(SD)	80.74 ± 35.77	118.80 ± 49.02	100.63 ± 47.13	<0.001
L4 HU, mean(SD)	79.19 ± 38.79	119.35 ± 50.99	100.18 ± 49.71	<0.001
L5 HU, mean(SD)	91.86 ± 41.31	132.51 ± 53.73	113.1 ± 52.21	<0.001
Hypertension				0.059
No	43(45%)	61(59%)	104(52%)	
Yes	52(55%)	43(41%)	95(48%)	
Diabetes				0.178
No	65(68%)	80(77%)	145(73%)	
Yes	30(32%)	24(23%)	54(27%)	
Smoking Status				0.504
No	81(85%)	92(88%)	173(86.9%)	
Yes	14(15%)	12(12%)	26(13.1%)	
Alcohol use				0.486
No	85(89%)	96(92%)	181(91%)	
Yes	10(11%)	8(8%)	18(9%)	
Back pain				<0.001
No	9(9%)	31(30%)	40(20%)	
Yes	86(91%)	73(70%)	159(80%)	

There were 199 participants. HU, Hounsfield Unit.

### Endplate deficit data

According to the presence of endplate defects, 199 patients were divided into an endplate defect group and a nonendplate defect group. Endplate defects were observed in 47.34% (n = 95) of the patients. In general, endplate defects were more common in elderly, female, and OP patients. Comparisons of HUs and other data between the two groups are shown in Table 1. We detected significant differences in age (70.6 ± 8.4 vs. 63.8 ± 9.5, p < 0.001), sex (male/female) (26/69 vs. 43/61, p < 0.05), BMI (23.8 ± 3.4 vs. 24.8 ± 3.4, p < 0.05), and total spine HUs (84.65 ± 35.49 vs. 124.86 ± 49.59, p < 0.001). In addition, we detected a significant correlation between low back pain and endplate defects (p < 0.001) (Table 1).

### Distribution of endplate defects

**FIGURE 3:**
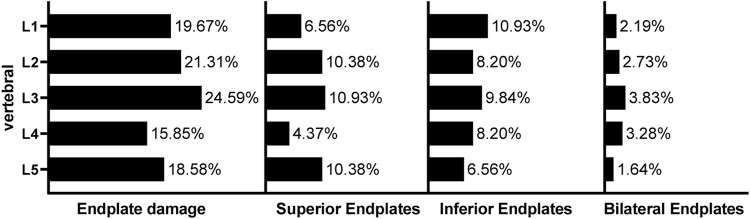
Incidence and distribution of lumbar endplate defects The data presented are prevalence rates and refer to the total sample studied for that particular vertebral level.

A total of 995 vertebral bodies from 199 patients were included, among which 183 (18.3%) had endplate defects. Among them, 78 (42.6%) patients had defects in the upper endplate, 80 (43.7%) patients had defects in the lower endplate, and 25 (13.7%) patients had bilateral endplate defects. Different types of endplate defects exhibited different distribution characteristics in different segments (Figure 3). The incidence of bilateral endplate defects in the L3 vertebral body was the highest (3.83%). In the upper lumbar spine, lower endplate defects were more common than upper endplate defects were, whereas upper endplate defects were more common in the lower lumbar spine (Table 2).

**TABLE 2:** Relationship between the dimensions of endplate defect on MRI and various parameters.

Parameters	Hounsfield unit		Sex-type		Level		Endplates	
	Low HU(HU ≤ 110) (n = 130)	Normal HU(HU > 110) (n = 53)	Males (n = 47)	Females (n = 136)	Upper lumbar (n = 120)	Lower lumbar (n = 63)	Superior (n = 78)	Inferior (n = 80)
Maximum width	10.9 ± 5.1	7.3 ± 4.8***	8.9 ± 4.6	10.2 ± 5.4	10.4 ± 5.5	8.8 ± 4.5	8.9 ± 5.1	10.0 ± 5.4
Maximum depth	4.8 ± 1.7	4.0 ± 1.7**	4.6 ± 1.6	4.5 ± 1.8	4.6 ± 1.8	4.4 ± 1.7	4.3 ± 1.6	4.6 ± 2.0
Standardized width	0.11 ± 0.08	0.08 ± 0.05**	0.08 ± 0.05	0.1 ± 0.08*	0.1 ± 0.07	0.09 ± 0.08	0.06 ± 0.02	0.09 ± 0.07**
Standardized depth	0.12 ± 0.06	0.09 ± 0.04**	0.1 ± 0.05	0.11 ± 0.06	0.11 ± 0.06	0.11 ± 0.05	0.09 ± 0.03	0.1 ± 0.05
Cumulative defect scores	0.23 ± 0.13	0.17 ± 0.09**	0.18 ± 0.1	0.22 ± 0.13*	0.21 ± 0.13	0.2 ± 0.12	0.16 ± 0.06	0.19 ± 0.11**

^a^ There were 183 endplates with defects. The “n values” represent the number of subjects from the total of 183 endplates.

^b^ Upper Lumbar: L1-L3; Lower Lumbar: L4-S1. mean values ± SD are noted, * p < 0.05, * p < 0.01, ***p < 0.001.

In addition, we measured the maximum widths and depths of the endplate defects on MRI. Comparisons of low HU group and normal HU group shown in Table 2. We detected significant differences in Maximum width (10.9 ± 5.1 vs. 7.3 ± 4.8, p < 0.001), Maximum depth (4.8 ± 1.7 vs. 4.0 ± 1.7, p < 0.01), Standardized width (0.11 ± 0.08 vs. 0.08 ± 0.05, p < 0.01), Standardized depth (0.12 ± 0.06 vs. 0.09 ± 0.04, p < 0.01), and Cumulative defect scores (0.23 ± 0.13 vs. 0.17 ± 0.09, p < 0.01). This indicates that the low HU group has a larger endplate defect compared with the normal group. We also found that female patients had larger endplate defect widths after normalization (standardized width, cumulative defect scores, p < 0.05), and the lower endplates had larger standardized defect widths and cumulative defect scores than did the upper endplates (p < 0.01).

**FIGURE 4:**
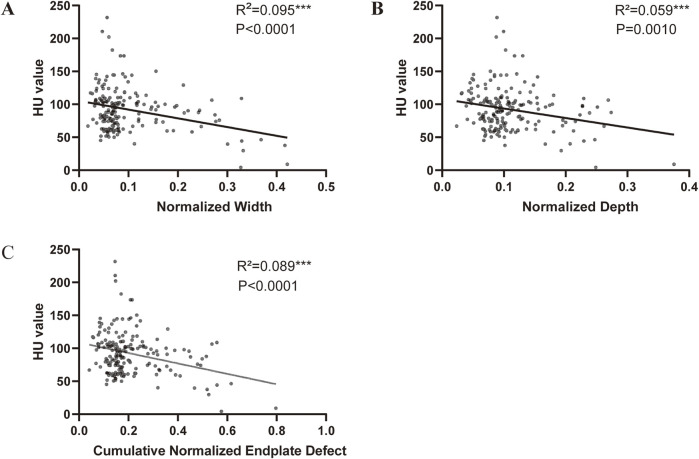
Correlation between endplate defect size and HU value **(A)** Linear correlation between HU value and width of endplate defect. **(B)** Linear correlation between HU value and depth of endplate defect. **(C)** Linear correlation between HU value and cumulative endplate defect score of endplate defect.

Scatter plots and linear regression analysis revealed that the normalized defect width, normalized defect depth, and cumulative defect scores were significantly negatively correlated with the HU value (Figure 4).

### Relationship between endplate defects and OP

To further clarify the relationship between endplate defects and lumbar BMD, we used logistic regression to analyse the relationships between endplate defects and age, sex, BMI, total spine HUs, hypertension, diabetes, smoking status, and alcohol use. After the effects of hypertension, diabetes, smoking status, and alcohol use were corrected for, there were statistically significant differences between endplate defects and age (OR = 1.0, p = 0.042) and total spine HUs (OR = 0.98, p = 0.001) (Table 3).

**TABLE 3:** Associations between the total Endplate Scores with the HU Value: results from univariate and multivariate logistic regression.

Category	Univariate association		Mulitivariate association	
	OR(95%CI)	p value	OR(95%CI)	p value
Age	1.1(1.1-1.2)	<0.001	1.0(1.0-1.1)	0.042
Sex				
male	Reference	—	Reference	—
female	1.9(1.0-3.4)	0.04	1.4(0.7-2.8)	0.328
BMI	0.9(0.8-1.0)	0.047	0.9(0.9-1.0)	0.268
Total spine HU	0.98(0.97-0.99)	0.001	0.98(0.97-0.99)	0.001
Hypertension				
No	Reference	—	Reference	—
Yes	1.7(1.0-3.0)	0.06	1.2(0.6-2.4)	0.511
Diabetes				
No	Reference	—		
Yes	1.5(0.8-2.9)	0.179		
Smoking Status				
No	Reference	—		
Yes	1.3(0.6-3.0)	0.505		
Alcohol use				
No	Reference	—		
Yes	0.7(0.3-1.9)	0.488		

There were 183 endplates with defects. HU, Hounsfield Unit; OR, odd ratio.

## Discussion

This study is the first to describe the morphology and distribution of endplate defects on MR images. Our study revealed that endplate defects were present in 47% of individuals and in 18% of vertebral bodies and that endplate defects were more common in individuals with OP, elderly individuals, and female patients (Table 1). With respect to the distribution of the spine, the endplate defects were mostly distributed in the upper lumbar spine (Figure 3). However, we found that the maximum width and depth of the vertebral endplate defect were not significantly correlated with the vertebral segment. Moreover, the mean age of patients with endplate defects was significantly greater than that of individuals with nonendplate defects, which was consistent with the study by Wang et al. (2012) This finding indicates that the depth of endplate defects increases with age and the progression of OP, which may be related to the fact that vertebral endplates become thinner and porous due to ageing, making it difficult for them to bear the corresponding mechanical load (Zehra et al., 2015). Additionally, the distribution of endplate defects in the upper lumbar spine was more extensive than that in the lower lumbar spine. This may be related to the greater thickness of the cortical bone in the lower lumbar spine than in the upper lumbar spine in order to withstand the increased load. (Edwards et al., 2001). Therefore, the lower lumbar spine had greater resistance to loading-induced endplate defects. In addition, compared with the lower lumbar spine, the upper lumbar spine participates more in rotational movement, the rotational movement puts a great deal of pressure on the endplate, and the repetitive rotational movement creates a tiny crack in the centre of the endplate cartilage, which becomes the weak part of the endplate; then, the crack dilates to form an endplate defect. (Gracovetsky and Farfan, 1986). In addition, some scholars believe that the inferior vertebral body has a larger cross-sectional area; during the period of axial loading, the mechanical stress is inversely proportional to the cross-sectional area of the vertebral body. (Duan et al., 2001). This may be another reason for the low incidence of lower vertebral endplate defects. The incidence of upper lumbar endplate defects was greater than that of lower endplate defects. There is no convincing explanation for this at present. Some scholars have proposed that this may be related to the fact that in the early fourth week of embryonic life in the early vertebral body, the cells of the cranial side are arranged loosely, whereas the cells on the caudal side are arranged densely. (Bury 1995; Moore et al., 2013). Therefore, in the early life cycle, the mechanical strength of the lower half of the vertebral body is weaker than that of the upper half, and the lower endplate is more susceptible to damage. In the lower lumbar spine, the proportion of superior endplate defects was greater, which may be related to the thinner cranial endplates supported by the trabecular bone with lower density (Zhao et al., 2009).

Our study, which was based on MRI and CT examinations, was the first to analyse the relationship between the size of the vertebral endplate defect and vertebral BMD. By measuring the vertebral HU value while avoiding more examinations for the patient, and previous studies have shown that the HU value is more effective than DXA in measuring BMD because, compared with DXA, the HU value measurement method avoids the influence of facet joints of the vertebral body. (Muraki et al., 2004; Pu et al., 2024). In addition, the endplate defect information of the patient was obtained through MRI examination. Analysis revealed that the width and standardized score of the endplate defect were greater in the low-bone-volume group than in the high-bone-volume group. In addition, correlation analysis further confirmed that the bone mass of the vertebral body was negatively correlated with the depth and width of the lamina defect. This finding is similar to that of the study by Zhuang et al. (2021), Ferguson and Steffen (2003). The vertebral endplate is a thin layer of tissue located between the vertebral body and the intervertebral disc. It acts as the mechanical interface between the rigid bone and the elastic disc and plays an important role in the transfer and bearing of mechanical stress in the spine. However, the vertebral endplate also acts as the main channel of nutrient supply, and its thin and porous structure plays an important role in intervertebral disc material transport, nutrient metabolism, and maintenance of nucleus pulposus pressure. However, this structure also makes it more susceptible to damage under the action of mechanical stress. BMD is closely related to the bearing capacity of bone. With decreasing BMD, bone is more susceptible to microfractures under the action of mechanical stress. In OP patients, central endplate deformation is more prominent (Ferguson and Steffen, 2003; DISC, 1988); moreover, the vertebral endplates are thinner, and the trabecular bone under the endplates is sparser. This may be a cause of vertebral endplate defects caused by OP. To further understand other risk factors for endplate defects, we used univariate multivariate logistic analysis and found that there were statistically significant associations between the total score of the endplate defect and age and total spine HUs. Increased age may accelerate bone metabolism disorders. When OP occurs in aging individuals, the vertebral endplates become thinner, and eventually, the vertebral endplate strength decreases. The faster damage accumulation under continuous cyclic loading makes the vertebral endplates thinner. Microcracks are generated to reduce the bearing capacity of the endplate under pressure. In addition, with aging, disc degeneration accelerates, the strength of the fibrous ring decreases, and horizontal cracks and blood vessel penetration of the endplate appear in the centre of the intervertebral disc and cartilage endplates (DISC, 1988; Roberts et al., 2006; Burr and Radin, 2003; Turgut et al., 2003). The weak point on the endplate becomes a factor in the herniation of the nucleus pulposus across the endplate and towards the vertebral body, resulting in endplate injury. Our study also revealed that vertebral endplate defects are closely related to low back pain. This may be related to the fact that endplate defects are often accompanied by vertebral endplate microfractures and the aggregation of inflammatory factors (Roberts et al., 2006; Burr and Radin, 2003; Turgut et al., 2003).

Finally, we attempted to explain the possible mechanisms underlying the relationship between OP and endplate defects. When vertebral body OP occurs, macroscopically, the vertebral endplate becomes thinner, and the remodelling of microfractures causes the trabecular bone under the endplate to be disordered under cyclic loading, the porosity of the endplate surface increases, and a cavity in the endplate occurs. Endplate edge defects or the formation of bone redundancy further reduce the mechanical load-resisting capacity of the vertebral endplate and increase the susceptibility of the vertebral endplate to breakage (Xiao et al., 2018; Luo et al., 2015; Liu et al., 2019; Luo et al., 2013; Sun et al., 2021; Zhong et al., 2016; Li et al., 2017; Xiao et al., 2020). At the microscopic level, OP changes the local bone metabolism microenvironment; bone resorption by osteoclasts is greater than bone formation by osteoblasts, resulting in abnormal bone reconstruction. (Frost, 1990). Pharmacological experiments confirmed that when anti-OP drugs are used, abnormal bone reconstruction of the endplate is inhibited, thus maintaining the structural integrity of the original endplate (Turgut et al., 2003; Liu et al., 2019; Luo et al., 2013; Sun et al., 2021). In addition, OP may accelerate the vascularization of the vertebral endplate. Neovascularization leads to the infiltration of inflammatory factors, macrophages, and mast cells, which also generate large amounts of growth factors, cytokines and enzymes, thus accelerating the destruction of the vertebral endplate and reducing its ability to withstand loads (Turgut et al., 2003).

Our study has a few limitations. First, our research is a cross-sectional study and cannot explain the causal relationship between vertebral endplate defects and osteoporosis. Further prospective experiments are needed for further investigation. In addition our measurement of vertebral endplate defects was based on imaging data. Although the measurements were made by experienced radiologists and orthopaedic surgeons, there are still gaps compared to results of direct measurement on cadavers. Besides, although we used the HU value to reflect the vertebral BMD, which can effectively avoid the influence of sclerotic bone and osteophytes on the vertebral BMD in the DXA measurement results. However, its accuracy is still lower than that of Q-CT. In addition, we used the VAS score scale to collect information on patients’ back pain. However, this cannot rule out other confounding factors causing back pain. Therefore, a more precise back pain scale can be further designed in subsequent studies to further clarify the relationship between back pain and endplate defects.

## Conclusion

There is a correlation between OP and the size of the vertebral endplate defect, and the defect size increases with decreasing bone mass. In the population, vertebral endplate defects are more likely to occur in elderly individuals, females, and individuals with OP. With respect to the spinal structure, vertebral endplate defects are more likely to occur in the upper lumbar spine. Additionally, age and bone mass are the main factors associated with vertebral endplate defects.

## Data Availability

The raw data supporting the conclusions of this article will be made available by the authors, without undue reservation.
