# Supplementary material for: Correlation analysis of osteoporosis and vertebral endplate defects using CT and MRI imaging: a retrospective cross-sectional study
Source: Front Physiol. 2025 Sep 23;16:1649477. doi: 10.3389/fphys.2025.1649477 (PMC12500679; doi:10.3389/fphys.2025.1649477)

Supplementary material 1. Visual Analogue Scale used for assessment of back pain.

How would you rate your average back pain intensity over the last week?

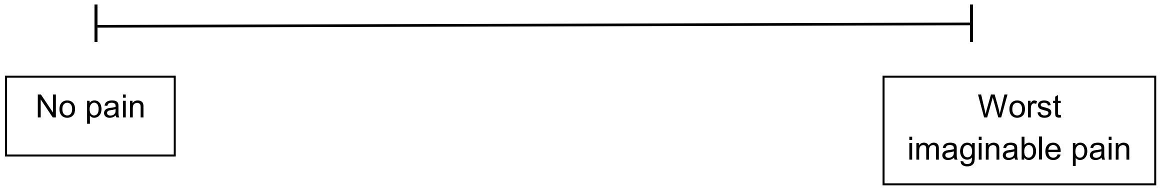

Supplement: Supplementary file 1 [file DataSheet1.pdf]
